# Supplementary material for: LXR pathway drives hormonal response intensity in polycystic ovary syndrome
Source: EMBO Mol Med. 2025 May 21;17(7):1666–85. doi: 10.1038/s44321-025-00251-1 (PMC12254376; doi:10.1038/s44321-025-00251-1)
Supplement: Supplementary file 1 — Appendix [file 44321_2025_251_MOESM1_ESM.pdf]

LXR Pathway Drives Hormonal Response Intensity in PCOS.

Sarah Dallel[1,2,3,4], Manon Despalles[1,2,3,4], Margaux Tore[1,2,3,4], Yoan Renaud[1,2,3,4], Ayhan Kocer[1,2,3,4], Christelle Damon-Soubeyrand[1,2,3,4], Pierre Pouchin[1,2,3,4], Caroline Vachias[1,2,3,4], Katia Boutourlinsky[1,2,3,4], Céline Gonthier-Gueret[1,2,3,4], Angélique De Haze[1,2,3,4], Phelipe Sanchez[1,2,3,4], Jean-Christophe Pointud[1,2], Erwan Bouchareb[1,2,3,4], Marine Vialat[1,2,3,4], Aurélie Lagarde[1,2,3,4], Cristina Gulunga[5], Laure Chaput[5], Aurélie Vega[5], Florence Brugnon[3],[5,6], Igor Tauveron[4], Amalia Trousson[1,2,3,4], Cyrille de Joussineau[1,2,3,4], Françoise Degoul[1,2,3,4], Laurent Morel[1,2,3,4], Jean Marc Lobaccaro[1,2,3,4], Salwan Maqdasy[1,2,3,4,$], Silvère Baron[1,2,3,4]*

APPENDIX

Appendix Table of Contents

1. Appendix Figure S1 Page 2
2. Appendix Figure S2 Page 3
3. Appendix Figure S3 Page 4
4. Appendix Figure S4 Page 5
5. Appendix Figure S5 Page 6
6. Appendix Figure S6 Page 7
7. Appendix Figure S7 Page 8
8. Appendix Figure S8 Page 9
9. Appendix Figure S9 Page 10
10. Appendix Figure S10 Page 11
11. Appendix Figure S11 Page 12
12. Appendix Figure S12 Page 13
13. Appendix Figure S13 Page 14
14. Appendix Figure S14 Page 15
15. Appendix Figure S15 Page 16
16. Appendix Table S1 Page 17

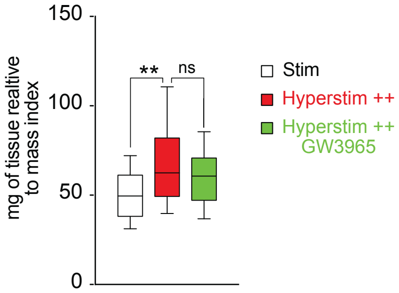

Appendix Figure S1: Ovary weights from wild-type mice that receive a single PMSG IP injection (7.5IU) and hCG IP injection (5IU) 46 hours later (Stim), or two IP injection (20IU) at hour 0 and 24 following a single hCG IP injection (10IU) (Hyperstimulation ++) and a similar protocol with additional GW3965 (20µg/mL) treatments 24 before starting the protocol and together with following PMSG/hCG IP injection (Hyperstimulation ++ GW3965). Significance determined by Mann and Whitney test. **P<0.01, n.s. non-significant.

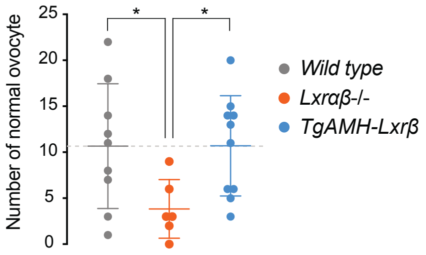

Appendix Figure S2: Number of normal oocytes retrieved in oviduct of of wild type, LXR DKO and TG-AMH-Lxrβ mice after ovulation following stimulation protocol. Significance determined by Mann and Whitney test. * P<0.05.

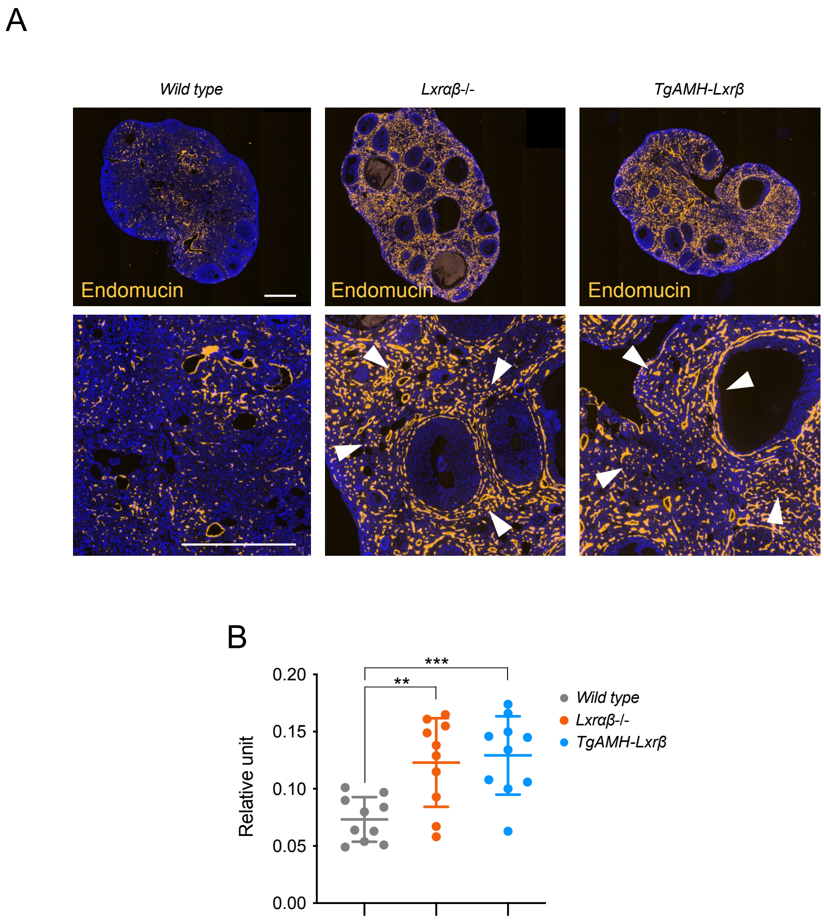

Appendix Figure S3: A - Wild type, LXR DKO and TG-AMH-Lxrβ ovaries 40hrs post-PMSG have been immunolabelled with Endomucin antibody (Scale bars = 500µm), white arrows indicated increased representation of vasculature network. B – Quantification of Evans blue dye accumulation in ovaries from wild type, LXR DKO and TG-AMH-Lxrβ. Significance determined by Mann and Whitney test. ** P<0.01, *** P<0.001.

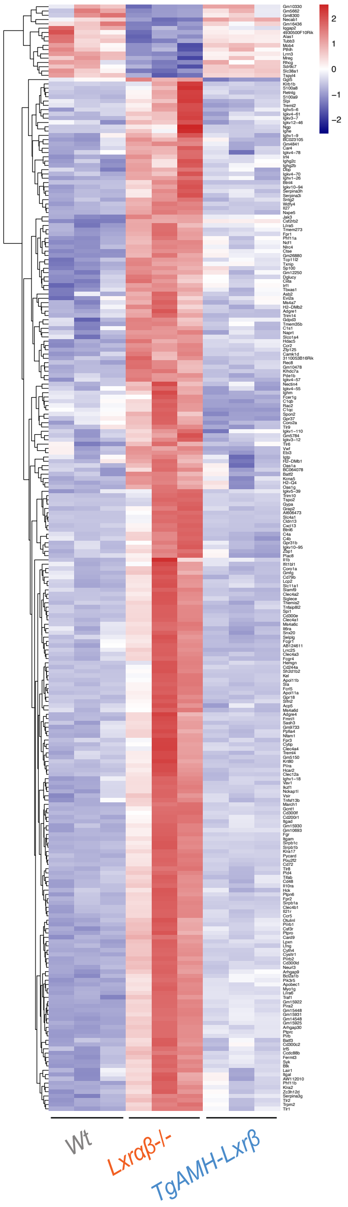

Appendix Figure S4: Heatmap of the 258 expression gene profiles identified comparing wild type versus LXR DKO and LXR DKO versus TG-AMH-Lxrβ dataset.

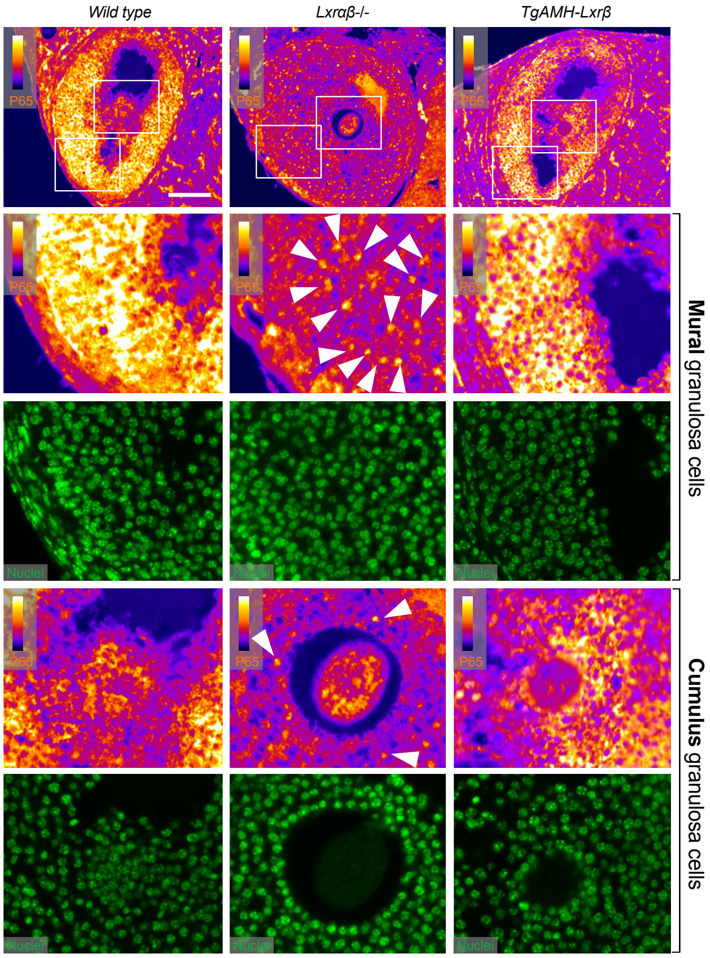

Appendix Figure S5: P65 immunodetection (fire scale), magnification of mural and cumulus granulosa compartment from each genotypes, nucleus staining (in green) is added to appreciate p65 localization. White arrows indicated nuclear translocation that Nucleus is mainly present in mural granulosa cell of LXR DKO ovaries compared to cumulus and absent in both wild type and TG-AMH-Lxrβ (Scale bars = 100µm).

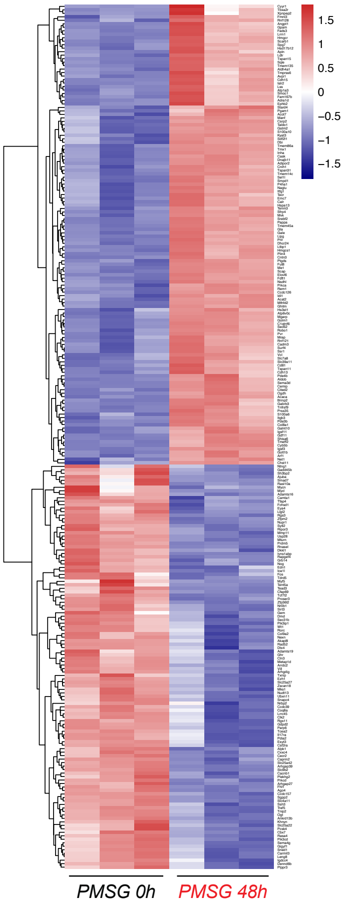

Appendix Figure S6: Heatmap of the PMSG-regulated gene expression profiles identified comparing dataset from granulosa cell collected on immature mice before and after PMSG stimulation for 48h [21].

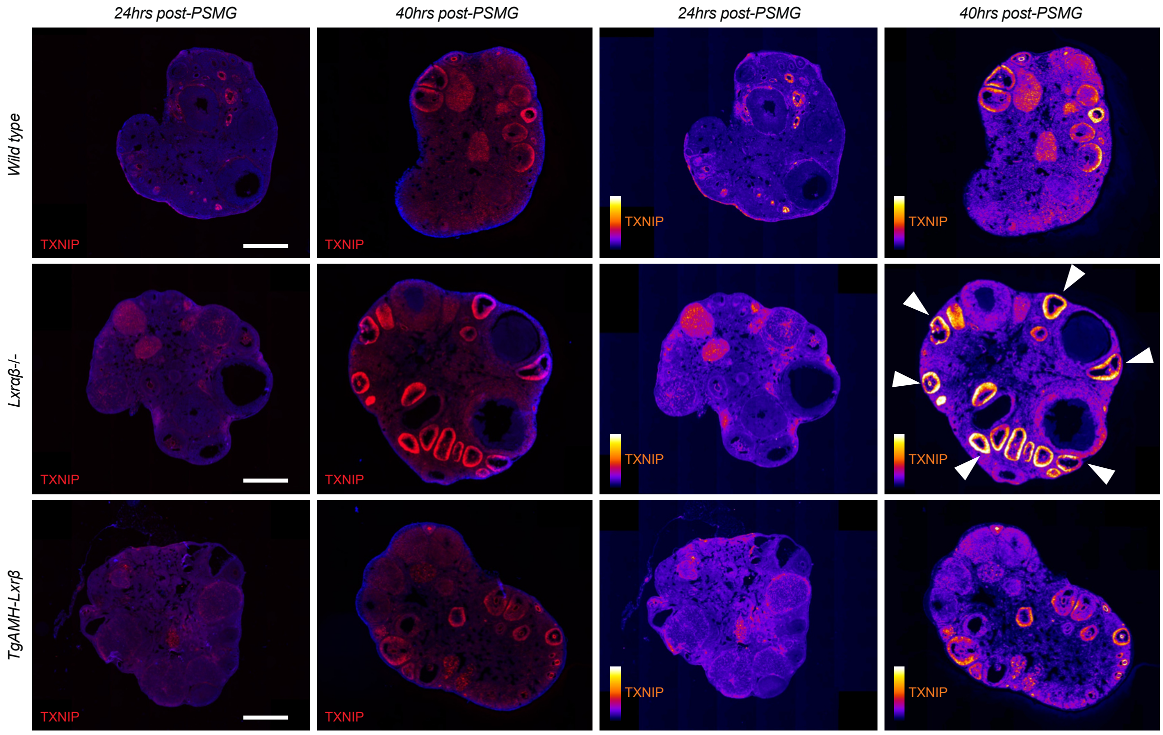

Appendix Figure S7: TXNIP immunodetection (fire scale) using wild type, LXR DKO and TG-AMH-Lxrβ ovaries after 24hrs or 40hrs post-PMSG. White arrows indicated overaccumulation of TXNIP in LXR DKO follicles (Scale bars = 500µm).

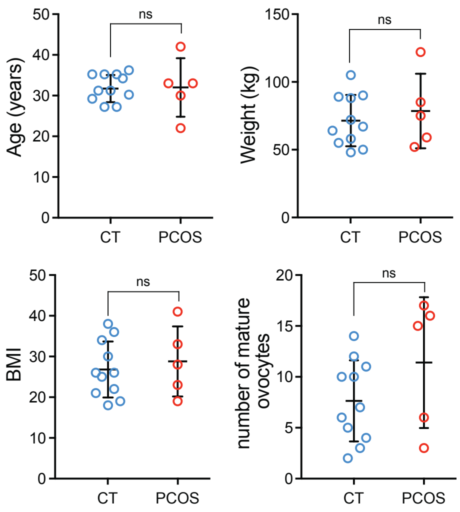

Appendix Figure S8: Statistical Analysis of human PCOS cohort parameters.

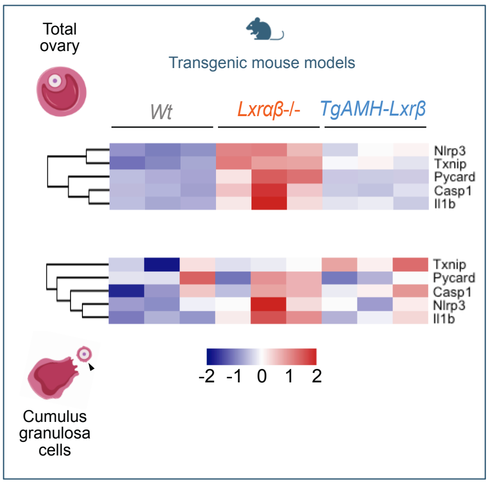

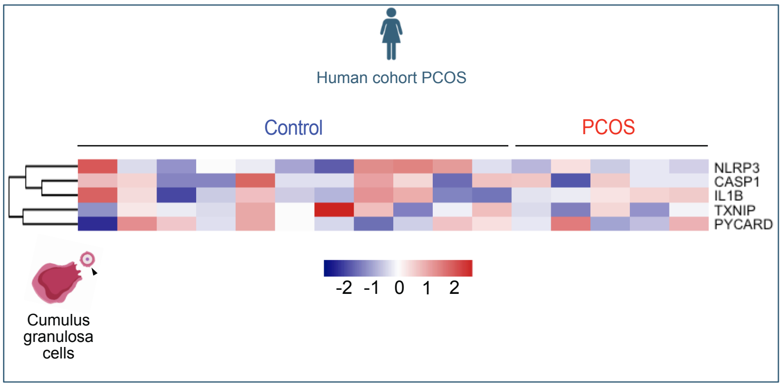

Appendix Figure S9: Heatmap of *Nlrp3*, *Txnip*, *Pycard (Asc)*, *Il1b* and *Casp1* gene expression for both total ovary and cumulus granulosa cell of wild type, LXR DKO and TG-AMH-Lxrβ mice and *NLRP3, TXNIP, PYCARD (ASC), IL1B* and *CASP1* for human PCOS cohort. Cumulus granulosa cell did not show differential DEGs either in mice models and human PCOS cohort.

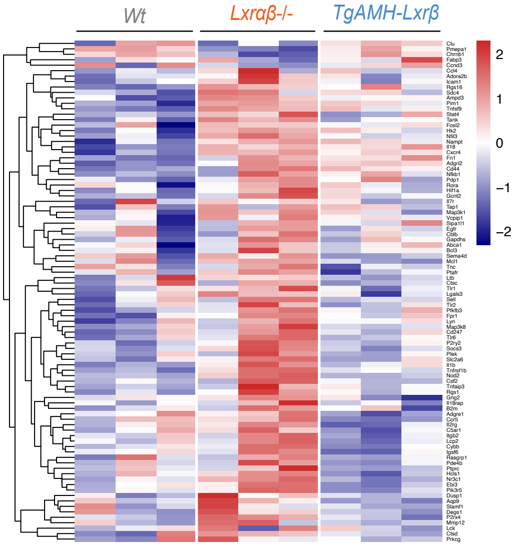

Appendix Figure S10: Heatmap of the HPS signature comparing wild-type, LXR DKO and TG-AMH-Lxrβ.

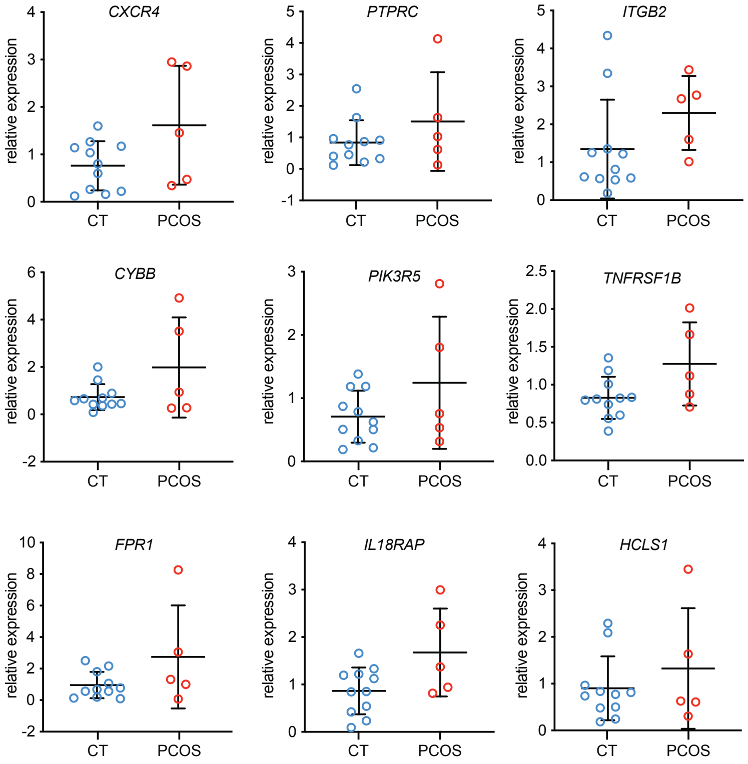

Appendix Figure S11: RT-qPCR analysis of *CXCR4, PTPRC, ITGB2, CYBB, PIK3R5, TNFRSF1B, FPR1, IL18RAP* and *HCLS1* gene expressions from human PCOS cohort.

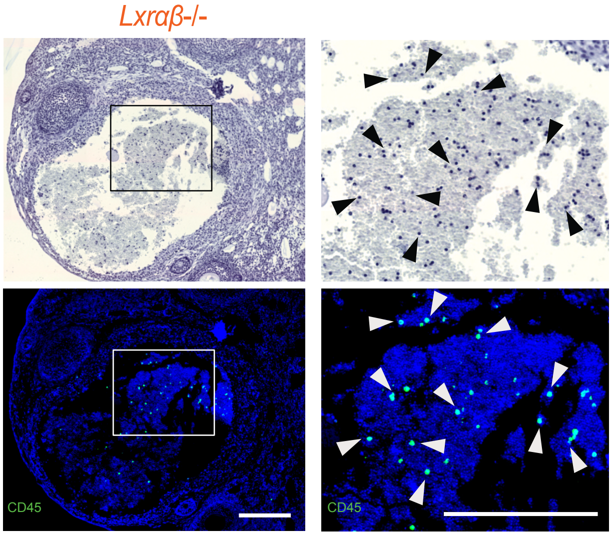

Appendix Figure S12: HE-staining (upper panel) and CD45 immunodetection (bottom panel) in LXR DKO hemorrhagic follicle in green. CD45+ cells have been observed in the antrum (arrows, scale bars = 100 µm).

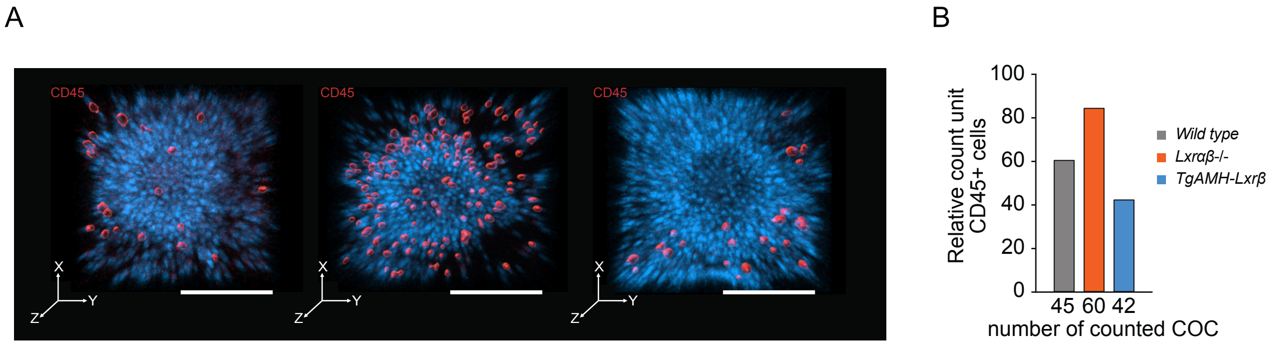

Appendix Figure S13: A - 3D confocal microscopy reconstruction of representative cumulus ovocyte complexes from wild type, LXR DKO and TG-AMH-Lxrβ mice post-ovulatory following hormonal stimulation (scale bar = 100 μm). B - Quantification of CD45-positive cells in Cumulus-Ovocytes Complex from each genotype.

A

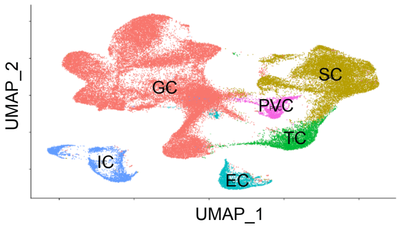

B

Cell type	Number	Percentage
Granulosa cell (GC)	37573	57.53%
Thecal cell (TC)	4307	6.59%
Stroma cell (SC)	14603	22.36%
Endothelial cell (EC)	3231	4.95%
Perivascular cell (PVC)	1924	2.95%
Immune cell (IC)	3673	5.62%

C

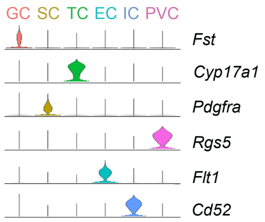

D

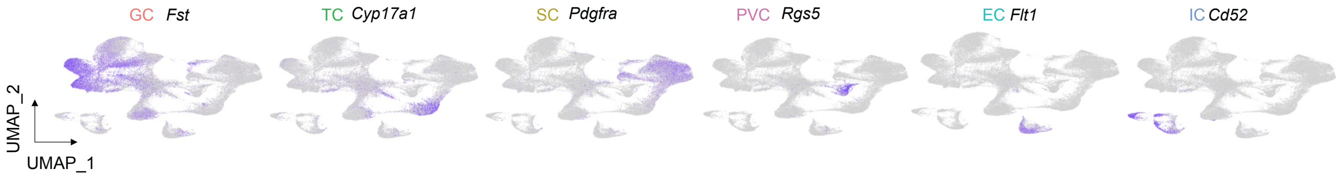

Appendix Figure S14: A - UMAP visualization of the six distinct cell clusters: granulosa cell (GC), thecal cells (TC), stroma cells (SC), endothelial cells (EC), perivascular cells (PVC), andi mmune cells (IC). B - Proportional representation of each cluster, C - Violin plots showing the expression profiles of specific markers for each cell cluster. D - Feature plots presenting the distribution of selected top genes within the ovarian cell clusters identified.

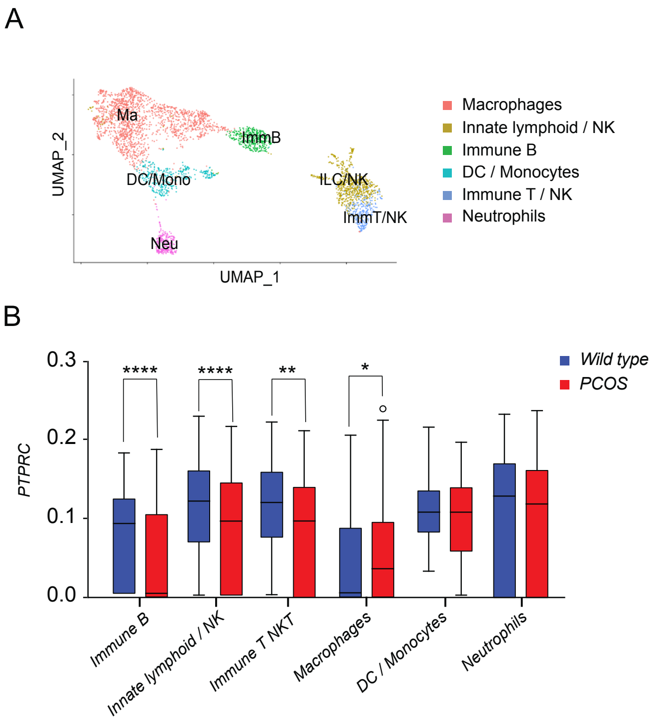

Appendix Figure S15: A – UMAP visualization classifying immune cells into six populations: macrophages (Ma), innate lymphoid / Natural Killer (ILC/NK), immune B (ImmB), dendritic / monocytes (DC/Mono), Immune T / Natural Killer (ImmT/NK) and neutrophils (Neu). B – Box plots showing the *PTPRC* expression in each cell cluster.

Appendix Table S1. Exact p-values obtained for the statistical comparisons performed in this study

Figure panel and comparison	Statistical test and p-value	Exact n
Figure 1C	Ordinary one-way ANOVA	
Stim vs. Hyperstim ++	<0.0001	14/18
Hyperstim ++ vs. Hyperstim ++ GW3965	0.0383	18/22
Stim vs. Hyperstim ++ GW3965	0.0360	14/22
Figure 1E	Mann Whitney test	
Cyp19a1 - Wild type vs. LXRαβ-/-	0.0022	6/6
Cyp19a1 - LXRαβ-/- vs. TgAMH-Lxrβ	0.0022	6/6
Fshr - Wild type vs. LXRαβ-/-	0.0152	6/6
Fshr - LXRαβ-/- vs. TgAMH-Lxrβ	0.0931	6/6
Inha - Wild type vs. LXRαβ-/-	0.0087	6/6
Inha - LXRαβ-/- vs. TgAMH-Lxrβ	0.0649	6/6
Figure 1F	Ordinary one-way ANOVA	
Wild type vs. LXRαβ-/-	0.0007	13/8
LXRαβ-/- vs. TgAMH-Lxrβ	0.0212	8/7
Figure 2B	Ordinary one-way ANOVA	
Follicular phase - Wild type vs. LXRαβ-/-	0.0117	22/16
Follicular phase - LXRαβ-/- vs. TgAMH-Lxrβ	0.0097	16/20
Ovulation - Wild type vs. LXRαβ-/-	<0.0001	18/20
Ovulation - LXRαβ-/- vs. TgAMH-Lxrβ	<0.0001	20/14
Luteal phase - Wild type vs. LXRαβ-/-	<0.0001	22/21
Luteal phase - LXRαβ-/- vs. TgAMH-Lxrβ	<0.0001	21/30
Figure 2G	Mann Whitney test	
Wild type vs. LXRαβ-/-	0.0002	10/10
LXRαβ-/- vs. TgAMH-Lxrβ	<0.0001	10/10
Figure 2L	Kolmogorov-Smirnov test	
NSG-Wild type vs. NSG	0.0001	12/8
NSG-LXRαβ-/- vs. NSG	<0.0001	14/8
NSG-TgAMH-Lxrβ vs. NSG	<0.0001	13/8
Figure 3C	Mann Whitney test	
Nfkbia - Wild type vs. LXRαβ-/-	0.02162	7/7
Nfkbia - LXRαβ-/- vs. TgAMH-Lxrβ	0.1061	7/5
Irf1 - Wild type vs. LXRαβ-/-	0.0070	7/7
Irf1 - LXRαβ-/- vs. TgAMH-Lxrβ	0.0480	7/5
Csf1 - Wild type vs. LXRαβ-/-	0.0262	7/7
Csf1 - LXRαβ-/- vs. TgAMH-Lxrβ	0.1061	7/5
Spi1 - Wild type vs. LXRαβ-/-	0.0023	7/7
Spi1 - LXRαβ-/- vs. TgAMH-Lxrβ	0.0480	7/5
Figure 5A	Mann Whitney test	
Wild type vs. LXRαβ-/-	0.0018	18/14
LXRαβ-/- vs. TgAMH-Lxrβ	0.0328	14/15
Figure 5C	Mann Whitney test	
CYP19A1 - DMSO vs. Forskolin	0.0022	6/6
TXNIP - DMSO vs. Forskolin	0.0043	5/6
Figure 5D	Mann Whitney test	
DMSO - Wild type vs. DMSO - LXRαβ-/-	0.0087	6/6
DMSO - LXRαβ-/- vs. DMSO - TgAMH-Lxrβ	0.0649	6/6
DMSO - Wild type vs .Forskolin - Wild type	0.0095	6/4
DMSO - LXRαβ-/- vs. Forskolin - LXRαβ-/-	0.0022	6/6
DMSO - TgAMH-Lxrβ vs. Forskolin - TgAMH-Lxrβ	0.0022	6/6

Forskolin - Wild type vs. Forskolin - LXRαβ-/-	0.0095	4/6
Forskolin - LXRαβ-/- vs. Forskolin - TgAMH-Lxrβ	0.0022	6/6
Figure 6B	Mann Whitney test	
Nlrp3 - Wild type vs. LXRαβ-/-	0.0018	17/16
Nlrp3 - LXRαβ-/- vs. TgAMH-Lxrβ	0.0555	16/15
Pycard - Wild type vs. LXRαβ-/-	0.0872	17/16
Pycard - LXRαβ-/- vs. TgAMH-Lxrβ	0.0933	16/15
Il1b - Wild type vs. LXRαβ-/-	0.0088	17/16
Il1b - LXRαβ-/- vs. TgAMH-Lxrβ	0.1370	16/15
Figure 6D	Spearman correlation	
Correl *Il1b/Nlrp3*	0.052	48
Correl *Il1b/Asc*	<0.0001	48
Correl *Nlrp3/Asc*	0.001	48
Figure 6E	Mann Whitney test	
DMSO vs. MCC950	0.0266	20/19
Figure 7E	Mann Whitney test	
Control vs. PCOS	0.0192	11/5
