# Supplementary figures and images for: LXR pathway drives hormonal response intensity in polycystic ovary syndrome
Source: EMBO Mol Med. 2025 May 21;17(7):1666–85. doi: 10.1038/s44321-025-00251-1 (PMC12254376; doi:10.1038/s44321-025-00251-1)

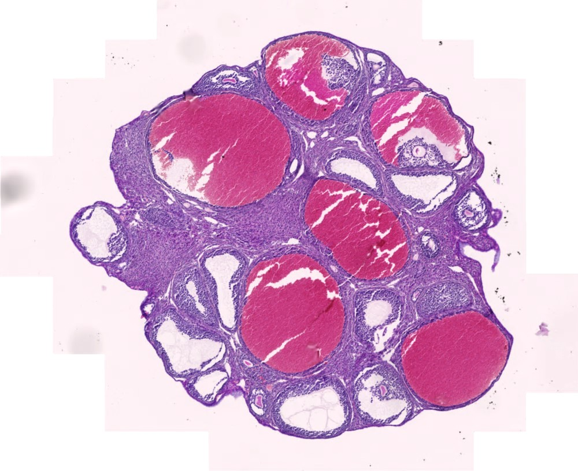

Supplement: Supplementary file 9 — Source data Fig. 1 [file 44321_2025_251_MOESM9_ESM.zip › Figure 1 /1B/Hyperstim++.tiff]

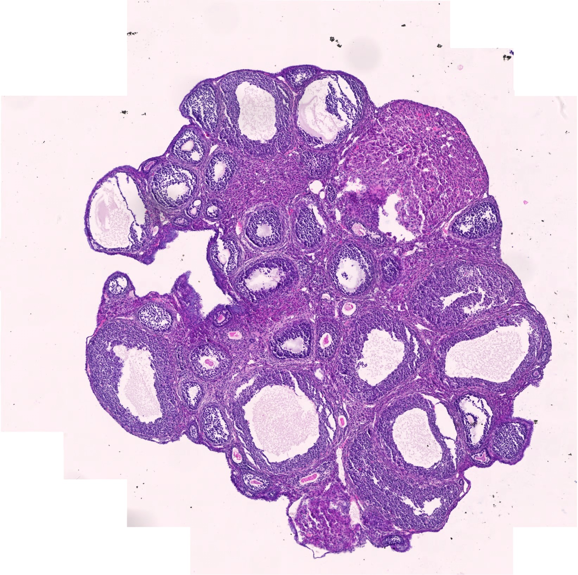

Supplement: Supplementary file 9 — Source data Fig. 1 [file 44321_2025_251_MOESM9_ESM.zip › Figure 1 /1B/Stim.tiff]

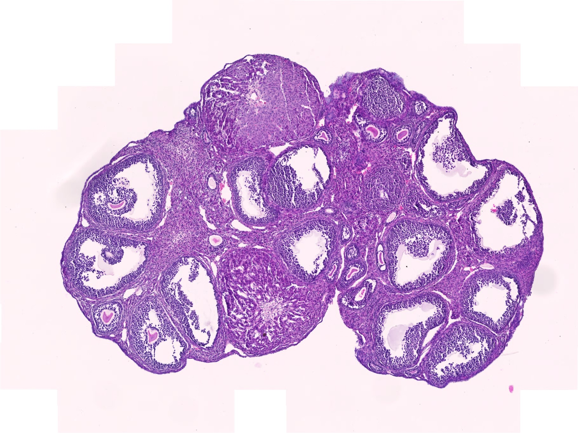

Supplement: Supplementary file 9 — Source data Fig. 1 [file 44321_2025_251_MOESM9_ESM.zip › Figure 1 /1B/Hyperstim++GW3965.tiff]

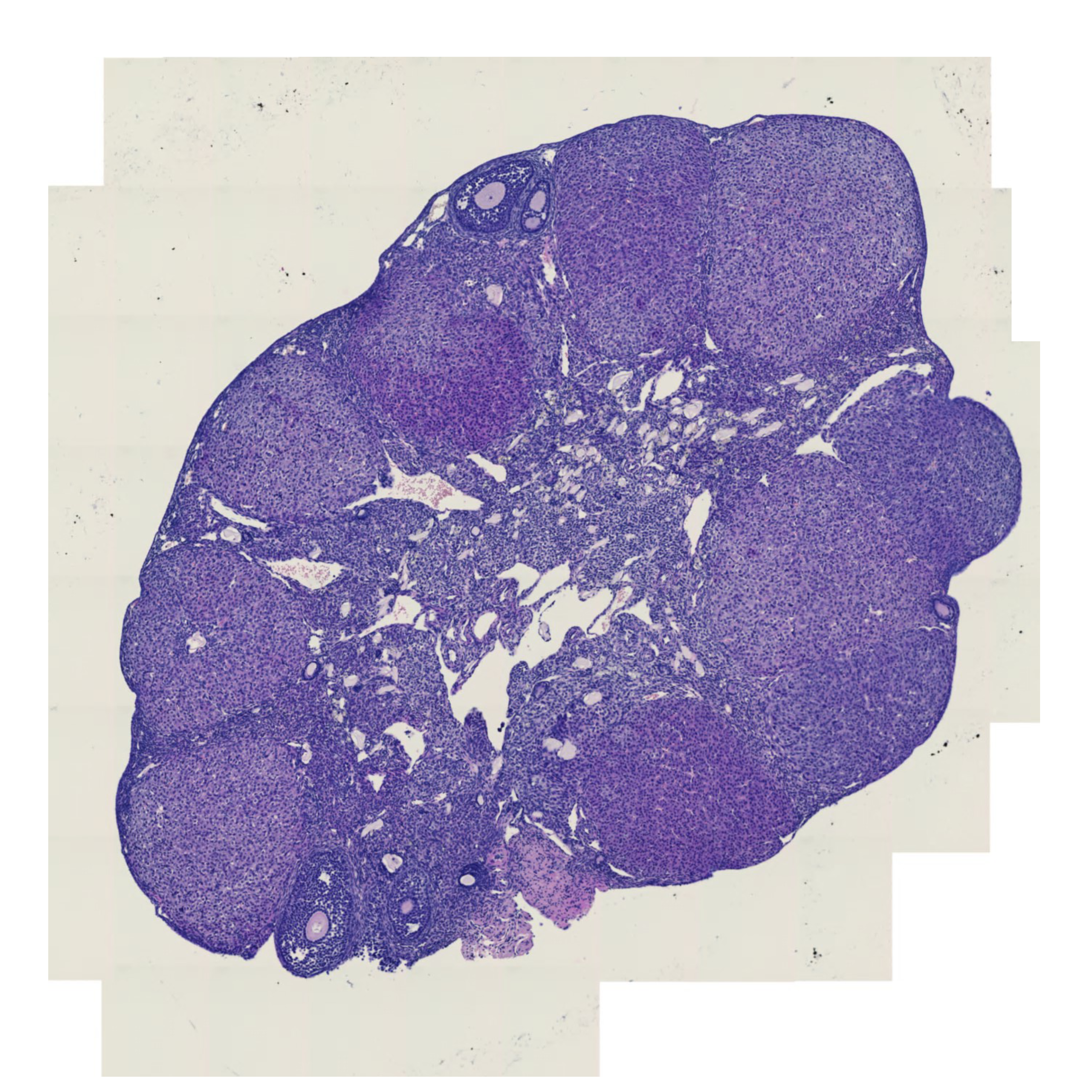

Supplement: Supplementary file 9 — Source data Fig. 1 [file 44321_2025_251_MOESM9_ESM.zip › Figure 1 /1D/TG AMH B.tiff]

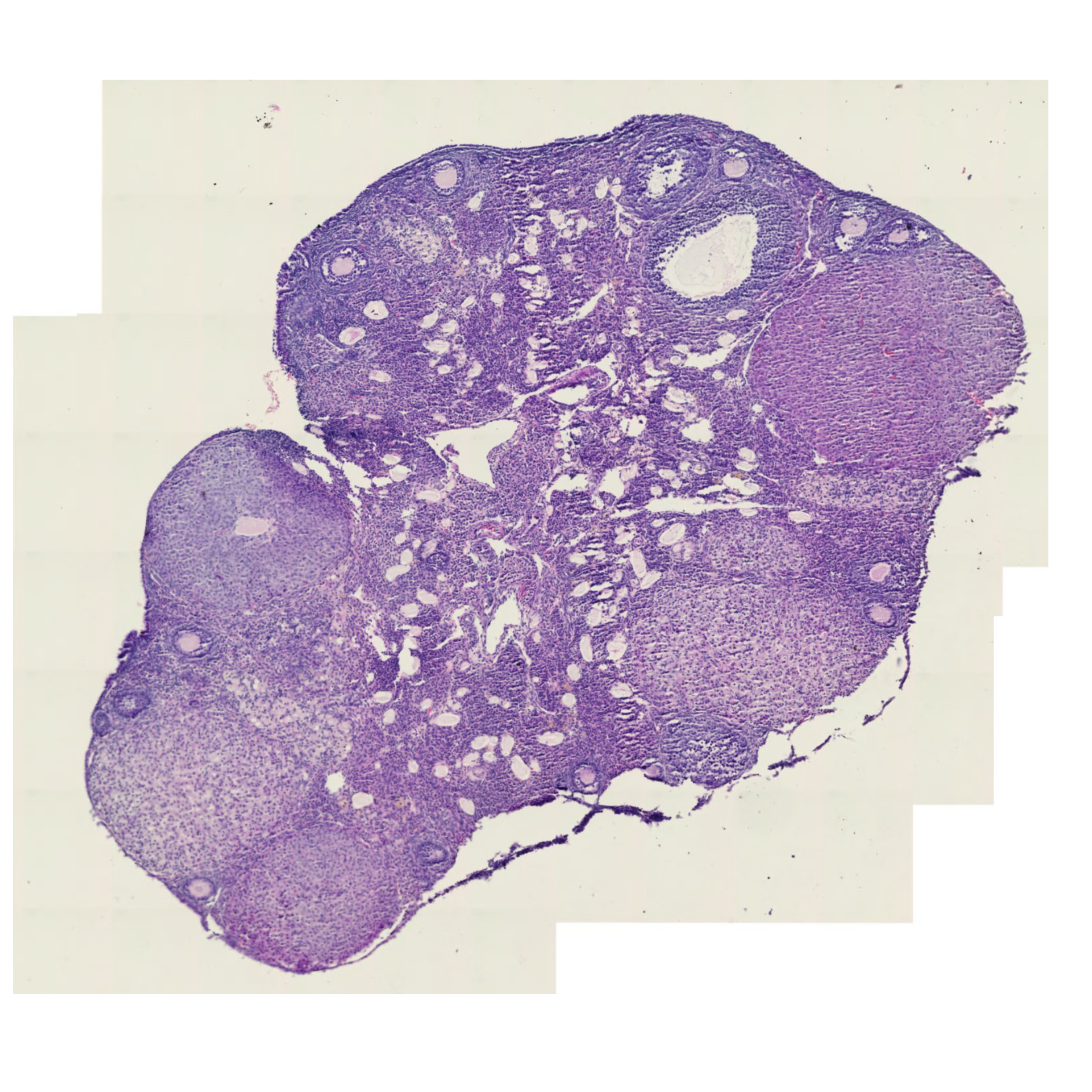

Supplement: Supplementary file 9 — Source data Fig. 1 [file 44321_2025_251_MOESM9_ESM.zip › Figure 1 /1D/WT.tiff]

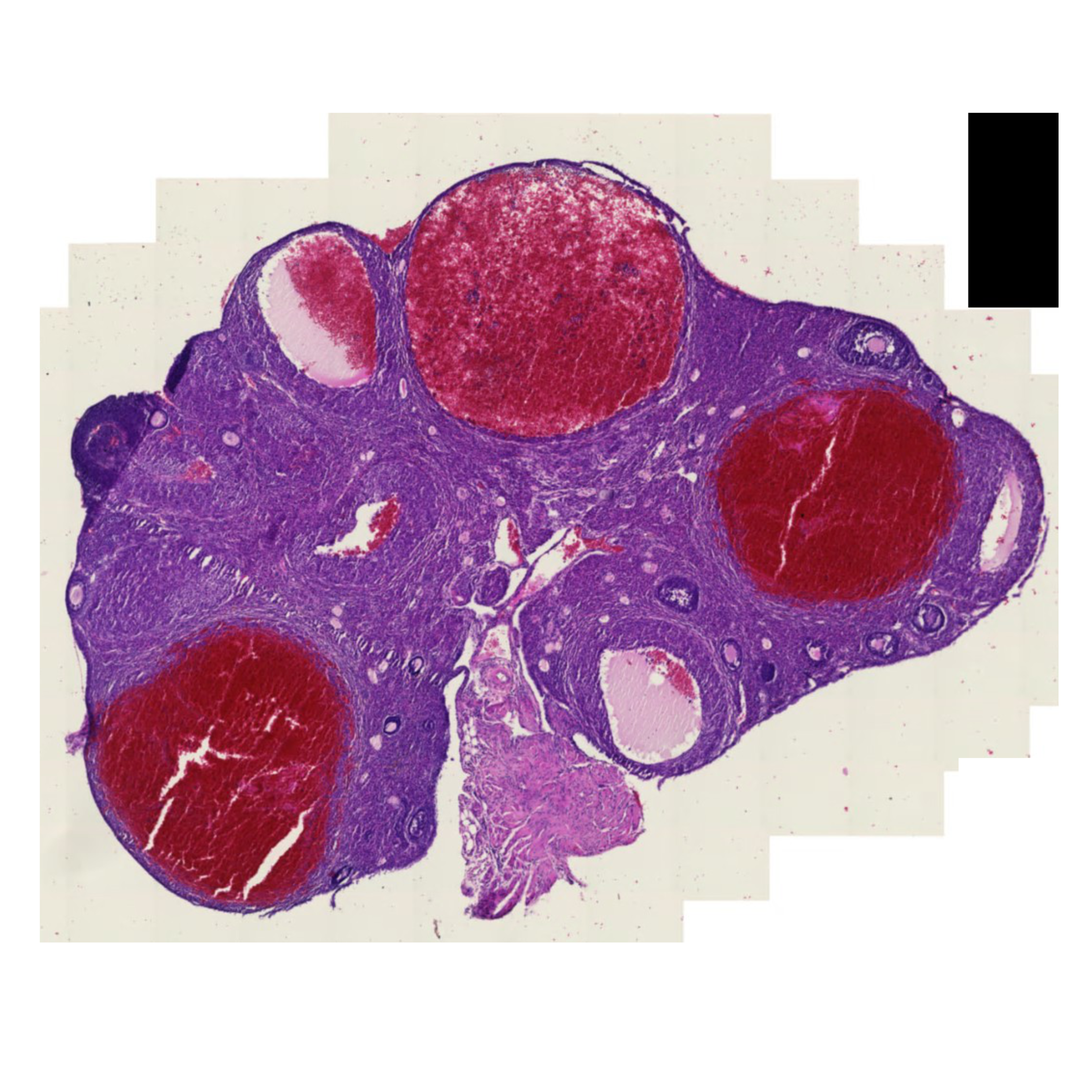

Supplement: Supplementary file 9 — Source data Fig. 1 [file 44321_2025_251_MOESM9_ESM.zip › Figure 1 /1D/LXR DKO.tiff]

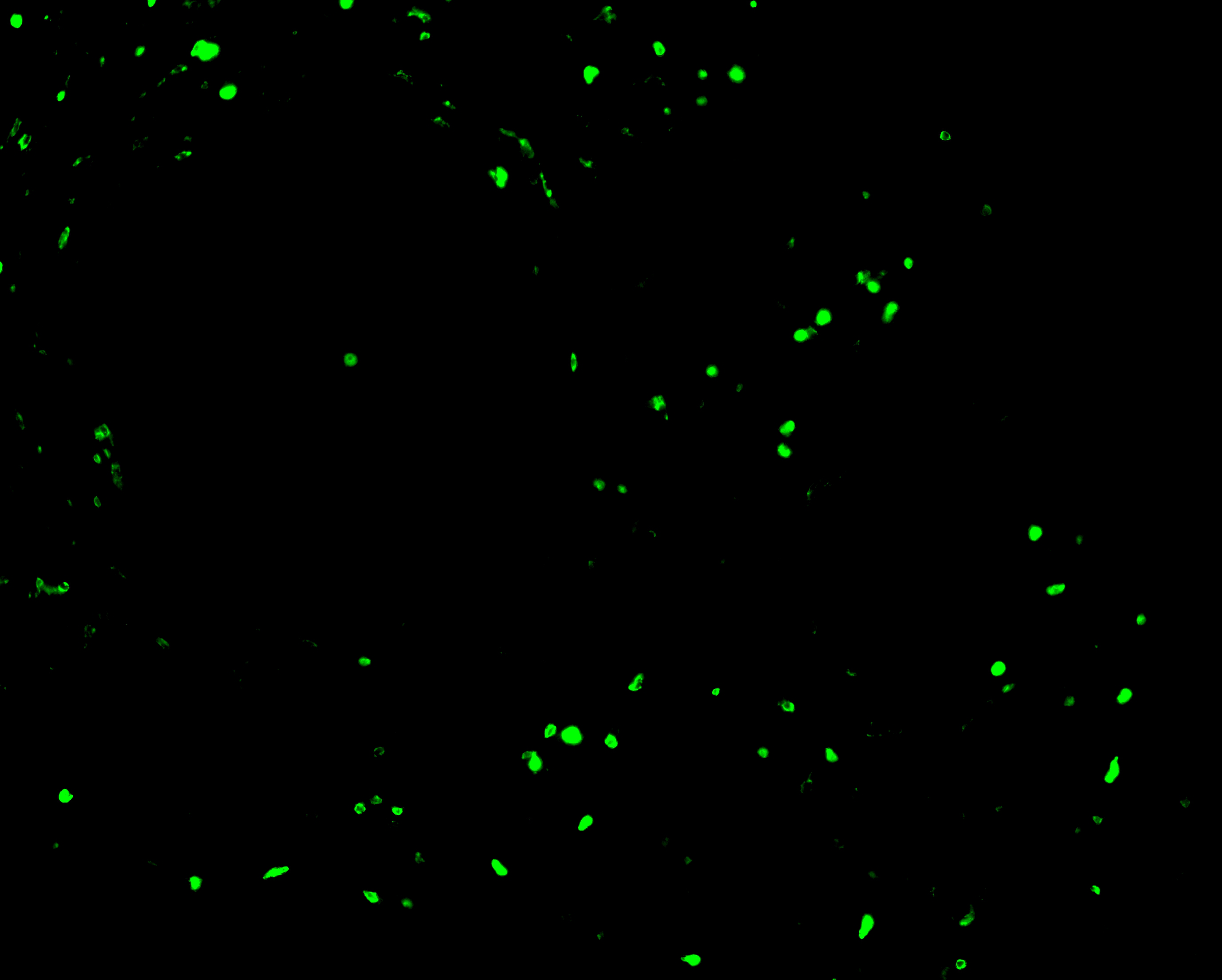

Supplement: Supplementary file 10 — Source data Fig. 2 [file 44321_2025_251_MOESM10_ESM.zip › Figure 2/2F/CD45 LXR DKO.tiff]

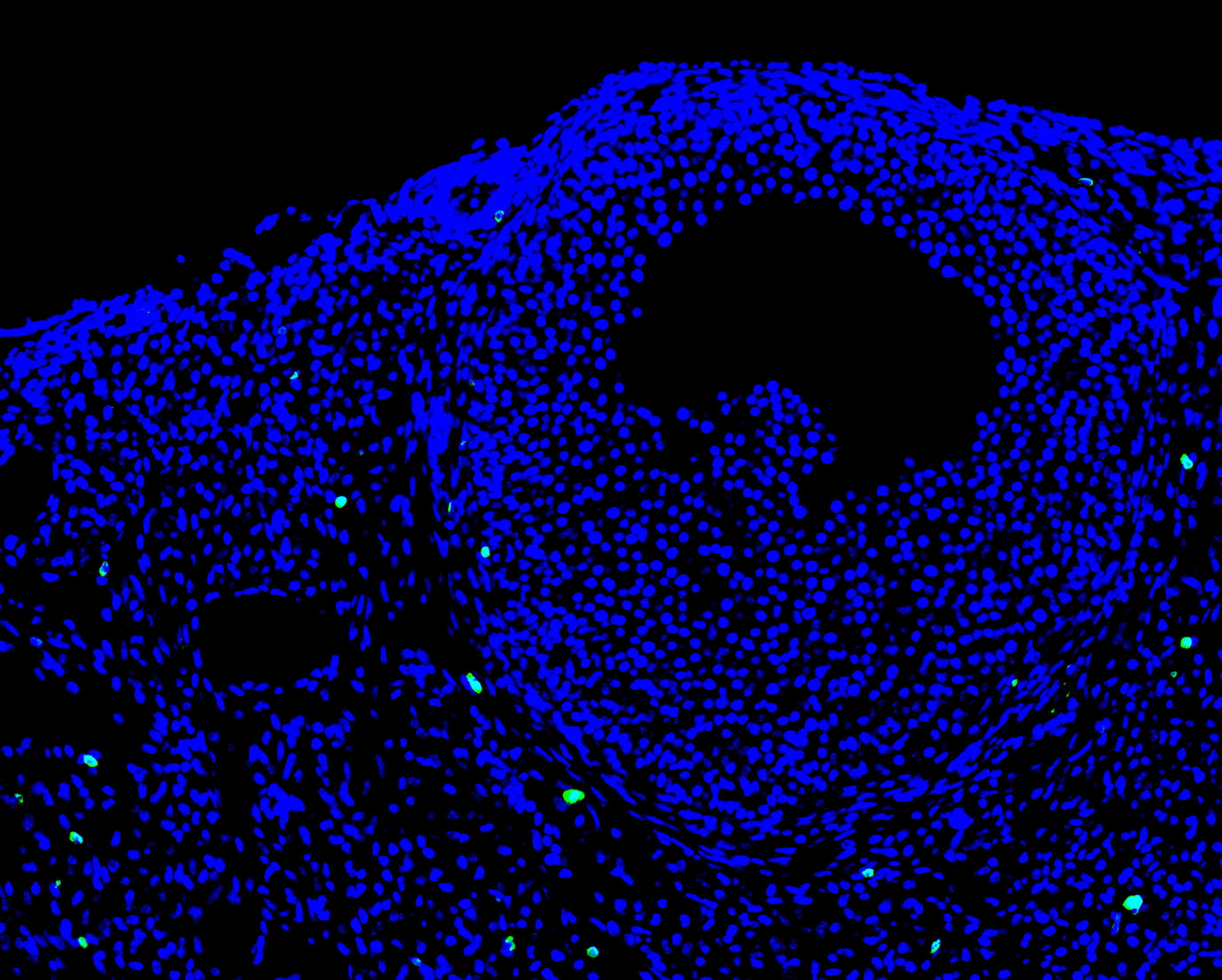

Supplement: Supplementary file 10 — Source data Fig. 2 [file 44321_2025_251_MOESM10_ESM.zip › Figure 2/2F/CD45 DAPI TG AMH B.tiff]

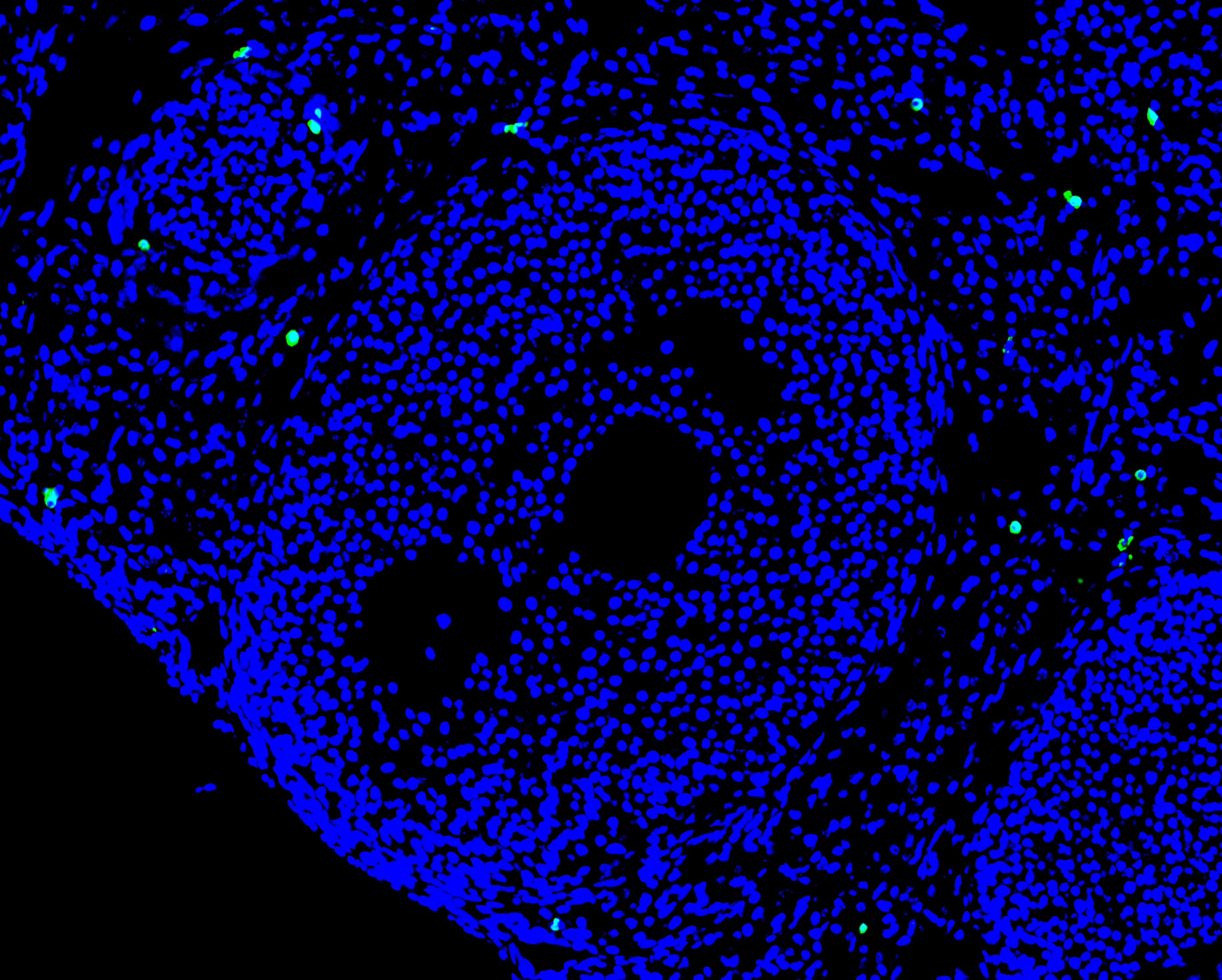

Supplement: Supplementary file 10 — Source data Fig. 2 [file 44321_2025_251_MOESM10_ESM.zip › Figure 2/2F/CD45 DAPI WT.tiff]

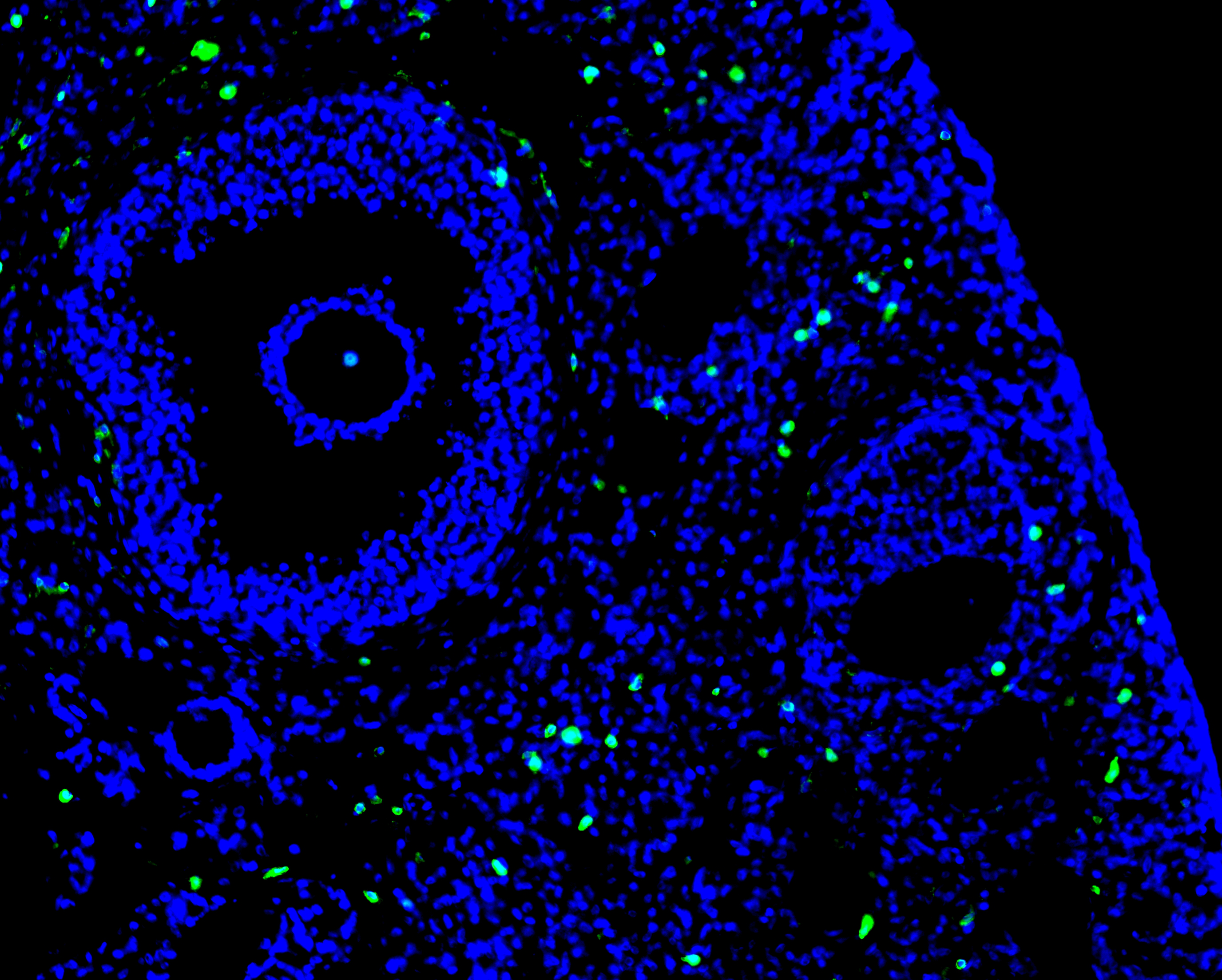

Supplement: Supplementary file 10 — Source data Fig. 2 [file 44321_2025_251_MOESM10_ESM.zip › Figure 2/2F/CD45 DAPI LXR DKO.tiff]

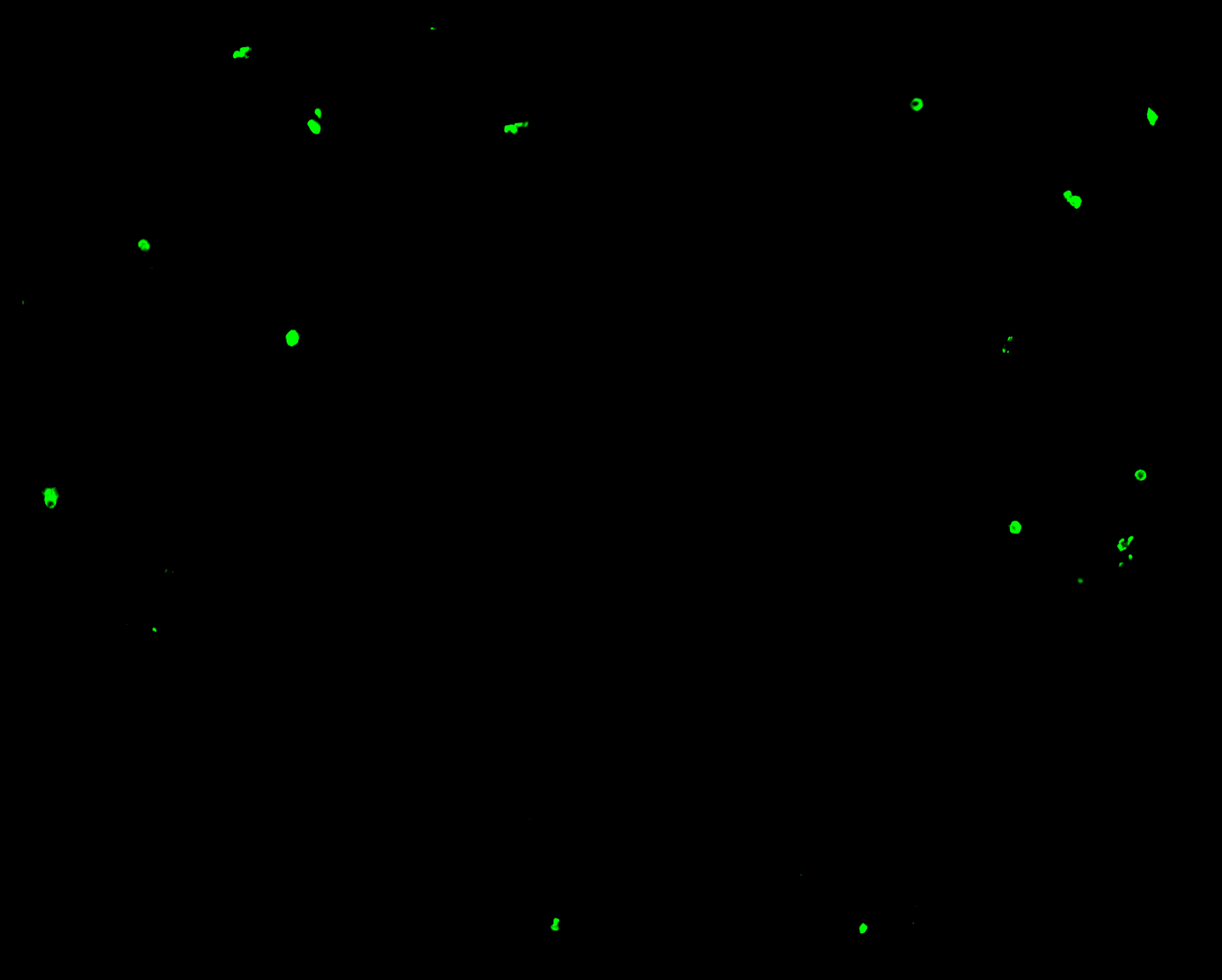

Supplement: Supplementary file 10 — Source data Fig. 2 [file 44321_2025_251_MOESM10_ESM.zip › Figure 2/2F/CD45 WT.tiff]

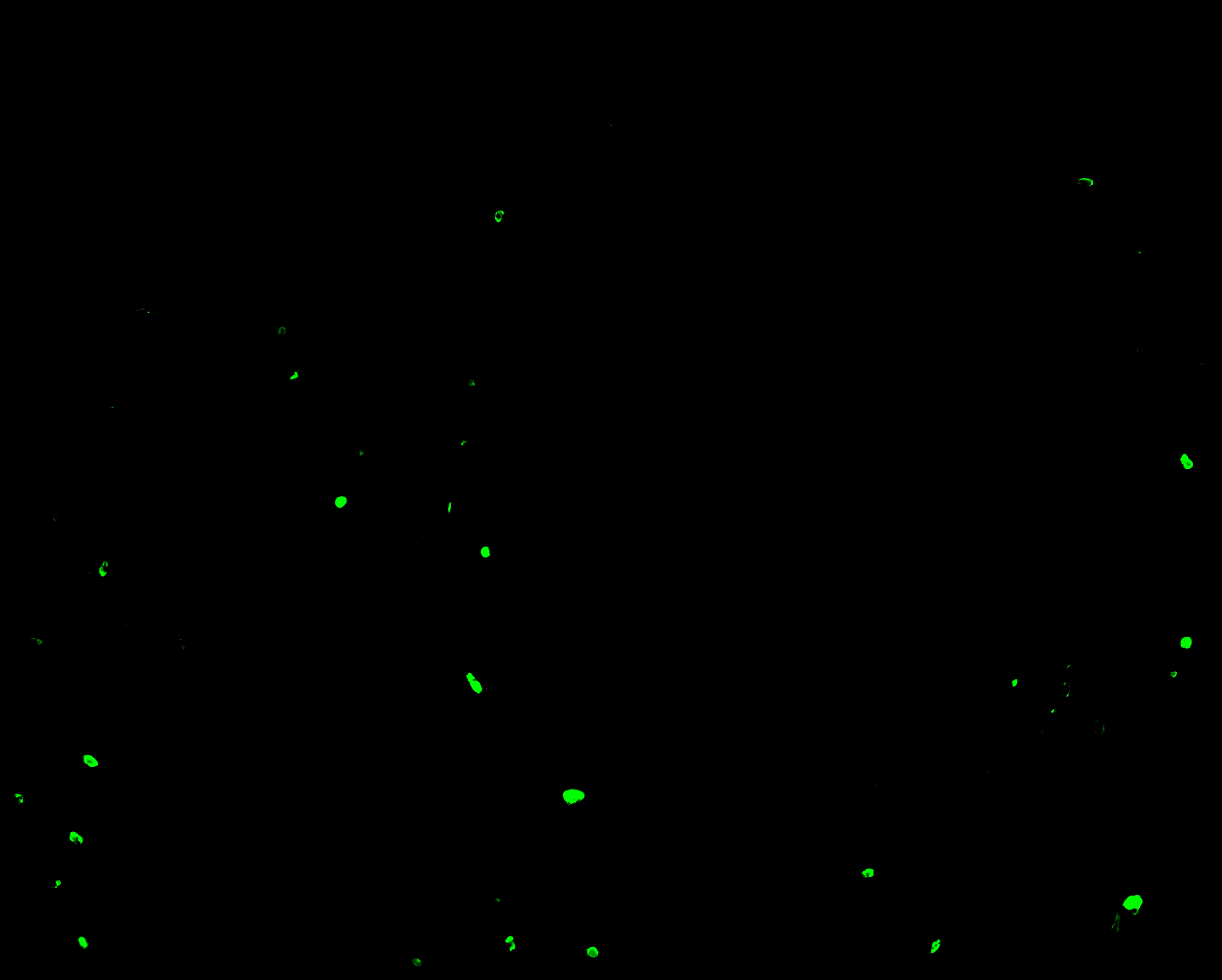

Supplement: Supplementary file 10 — Source data Fig. 2 [file 44321_2025_251_MOESM10_ESM.zip › Figure 2/2F/CD45 TG AMH B.tiff]

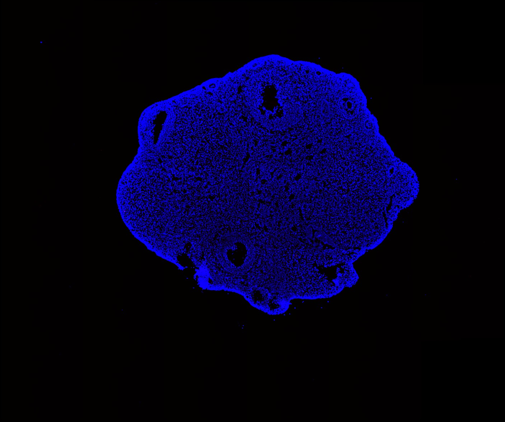

Supplement: Supplementary file 10 — Source data Fig. 2 [file 44321_2025_251_MOESM10_ESM.zip › Figure 2/2K/NSG MHC II.tiff]

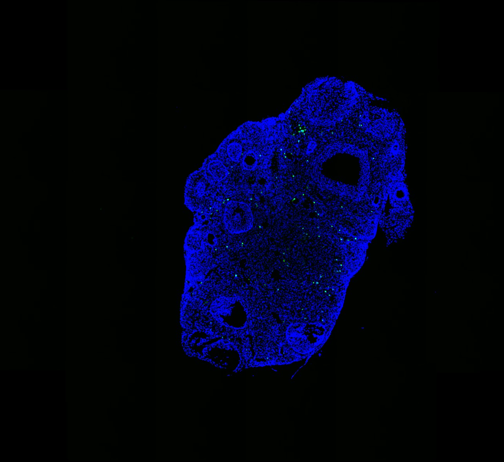

Supplement: Supplementary file 10 — Source data Fig. 2 [file 44321_2025_251_MOESM10_ESM.zip › Figure 2/2K/NSG-Wt CD45.tiff]

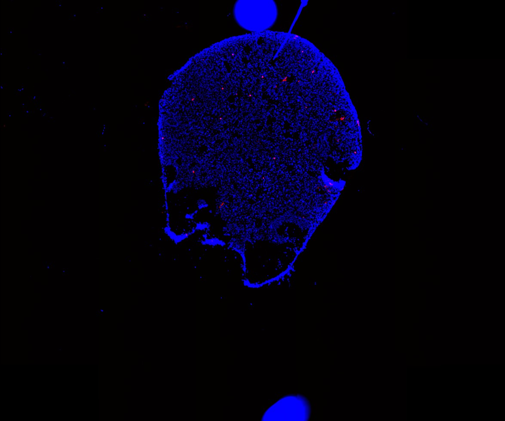

Supplement: Supplementary file 10 — Source data Fig. 2 [file 44321_2025_251_MOESM10_ESM.zip › Figure 2/2K/NSG-Wt MHC II.tiff]

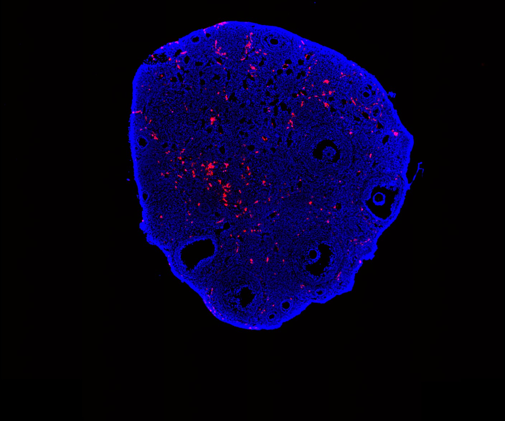

Supplement: Supplementary file 10 — Source data Fig. 2 [file 44321_2025_251_MOESM10_ESM.zip › Figure 2/2K/NSG-LXR DKO MHC II.tiff]

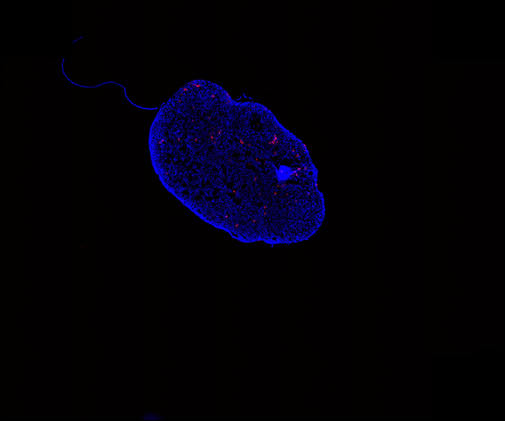

Supplement: Supplementary file 10 — Source data Fig. 2 [file 44321_2025_251_MOESM10_ESM.zip › Figure 2/2K/NSG-TG AMH B MHC II.tiff]

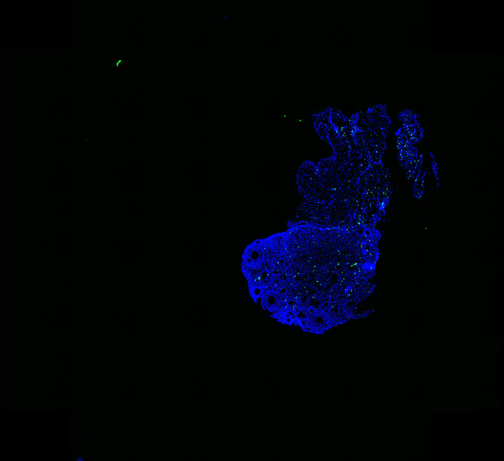

Supplement: Supplementary file 10 — Source data Fig. 2 [file 44321_2025_251_MOESM10_ESM.zip › Figure 2/2K/NSG-TG AMH B CD45.tiff]

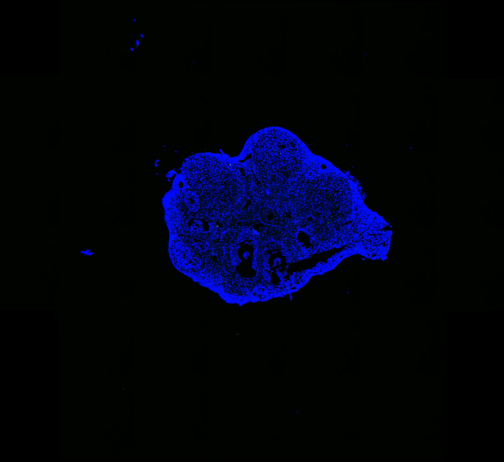

Supplement: Supplementary file 10 — Source data Fig. 2 [file 44321_2025_251_MOESM10_ESM.zip › Figure 2/2K/NSG CD45.tiff]

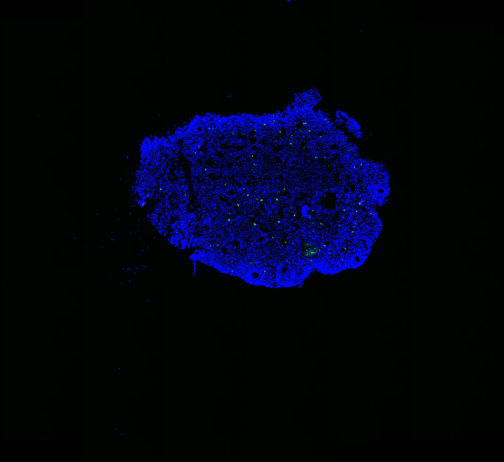

Supplement: Supplementary file 10 — Source data Fig. 2 [file 44321_2025_251_MOESM10_ESM.zip › Figure 2/2K/NSG-LXR DKO CD45.tiff]

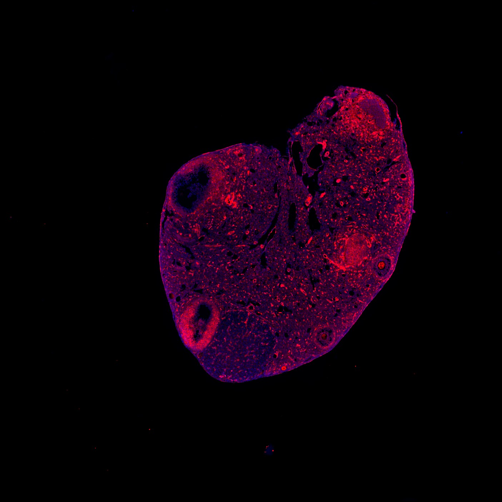

Supplement: Supplementary file 11 — Source data Fig. 3 [file 44321_2025_251_MOESM11_ESM.zip › Figure 3/3D/p65 WT.tiff]

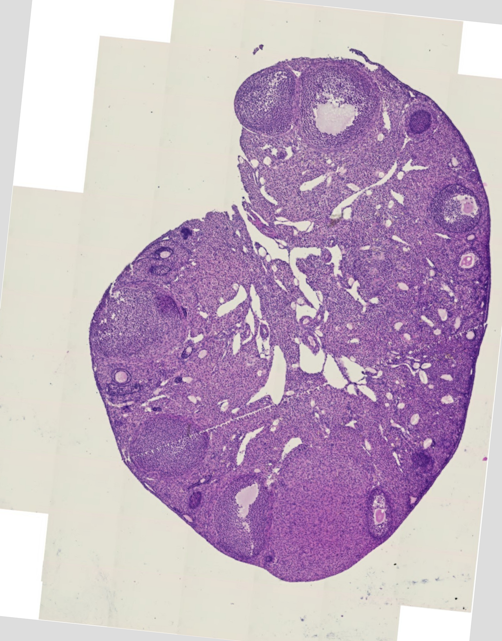

Supplement: Supplementary file 11 — Source data Fig. 3 [file 44321_2025_251_MOESM11_ESM.zip › Figure 3/3D/HE WT.tiff]

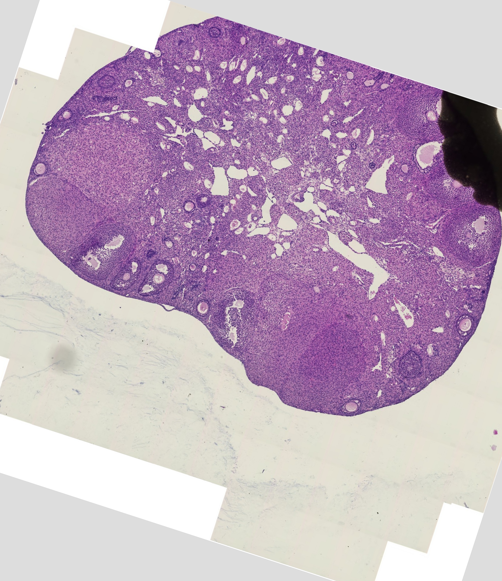

Supplement: Supplementary file 11 — Source data Fig. 3 [file 44321_2025_251_MOESM11_ESM.zip › Figure 3/3D/HE TG AMH B.tiff]

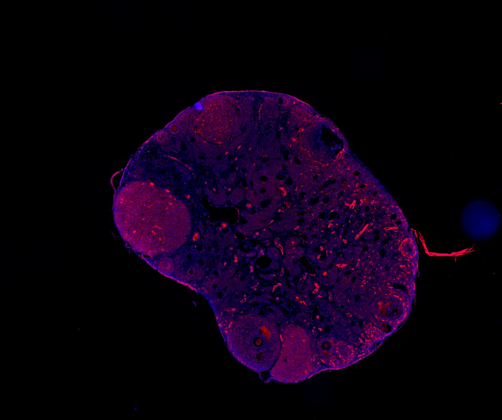

Supplement: Supplementary file 11 — Source data Fig. 3 [file 44321_2025_251_MOESM11_ESM.zip › Figure 3/3D/p65 LXR DKO.tiff]

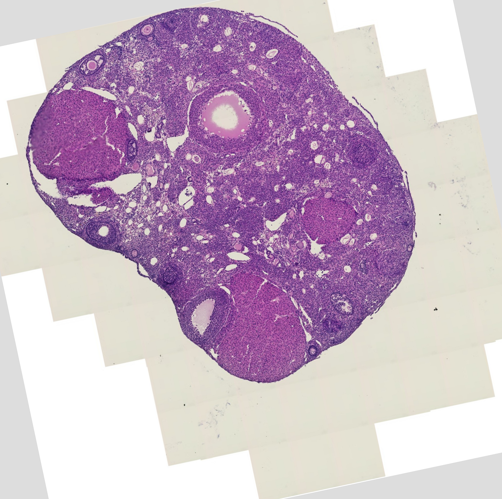

Supplement: Supplementary file 11 — Source data Fig. 3 [file 44321_2025_251_MOESM11_ESM.zip › Figure 3/3D/HE LXR DKO.tiff]

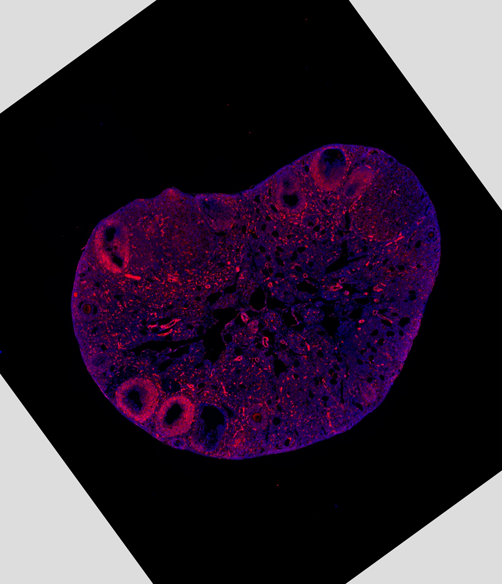

Supplement: Supplementary file 11 — Source data Fig. 3 [file 44321_2025_251_MOESM11_ESM.zip › Figure 3/3D/p65 TG AMH B.tiff]

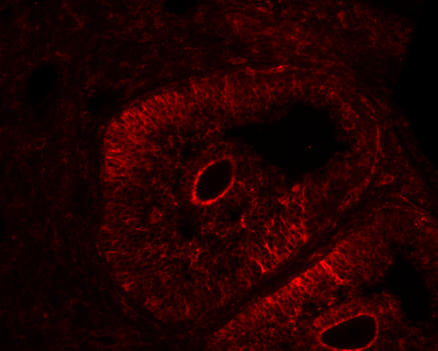

Supplement: Supplementary file 12 — Source data Fig. 5 [file 44321_2025_251_MOESM12_ESM.zip › Figure 5/5B/TXNIP TG AMH B.tiff]

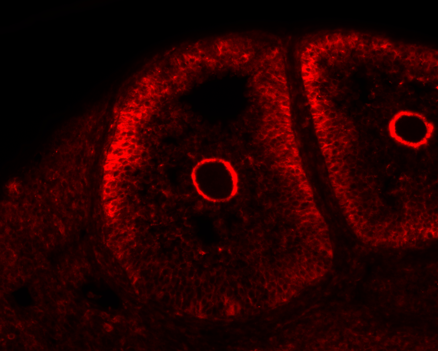

Supplement: Supplementary file 12 — Source data Fig. 5 [file 44321_2025_251_MOESM12_ESM.zip › Figure 5/5B/TXNIP WT.tiff]

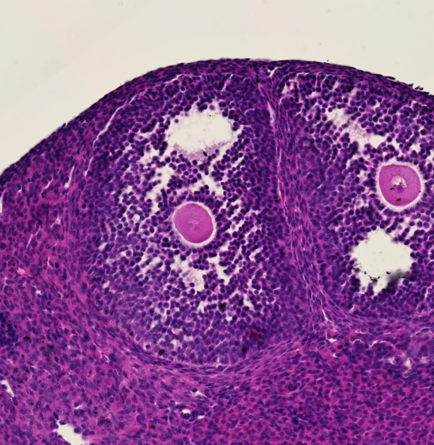

Supplement: Supplementary file 12 — Source data Fig. 5 [file 44321_2025_251_MOESM12_ESM.zip › Figure 5/5B/HE WT.tiff]

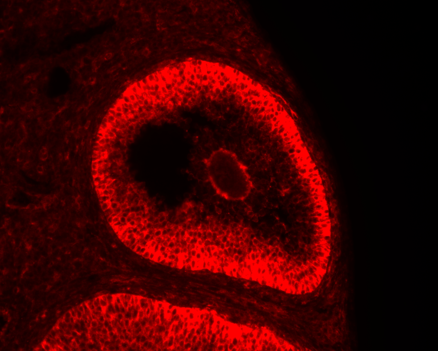

Supplement: Supplementary file 12 — Source data Fig. 5 [file 44321_2025_251_MOESM12_ESM.zip › Figure 5/5B/TXNIP LXR DKO.tiff]

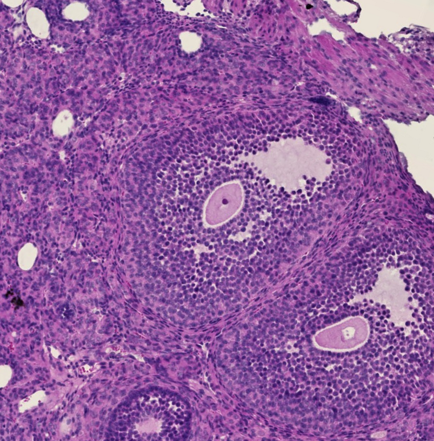

Supplement: Supplementary file 12 — Source data Fig. 5 [file 44321_2025_251_MOESM12_ESM.zip › Figure 5/5B/HE TG AMH B.tiff]

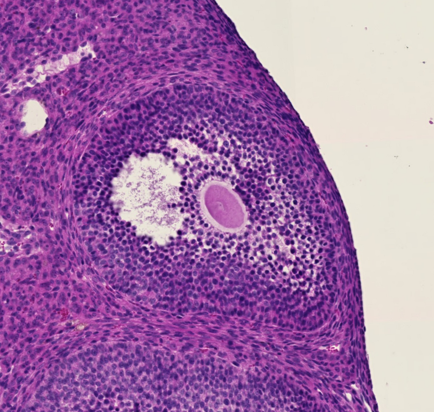

Supplement: Supplementary file 12 — Source data Fig. 5 [file 44321_2025_251_MOESM12_ESM.zip › Figure 5/5B/HE LXR DKO.tiff]

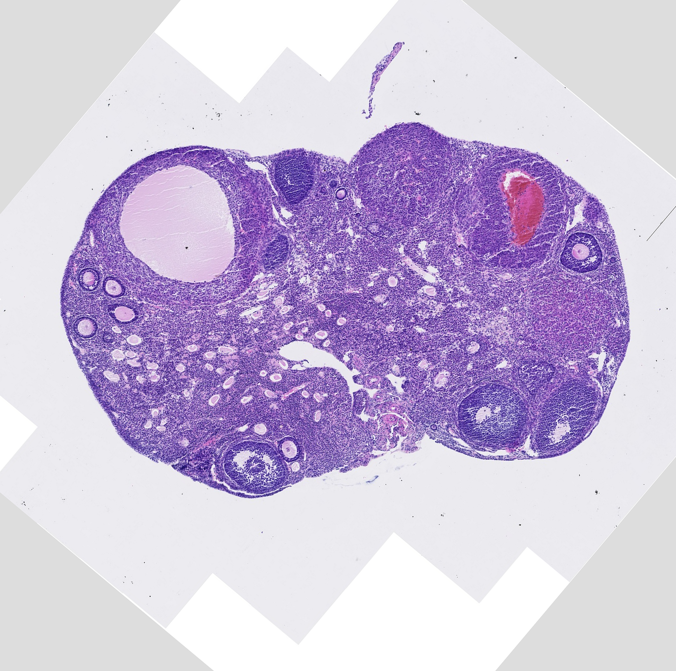

Supplement: Supplementary file 13 — Source data Fig. 6 [file 44321_2025_251_MOESM13_ESM.zip › Figure 6/6F/HE DKO LXR.tif.tiff]

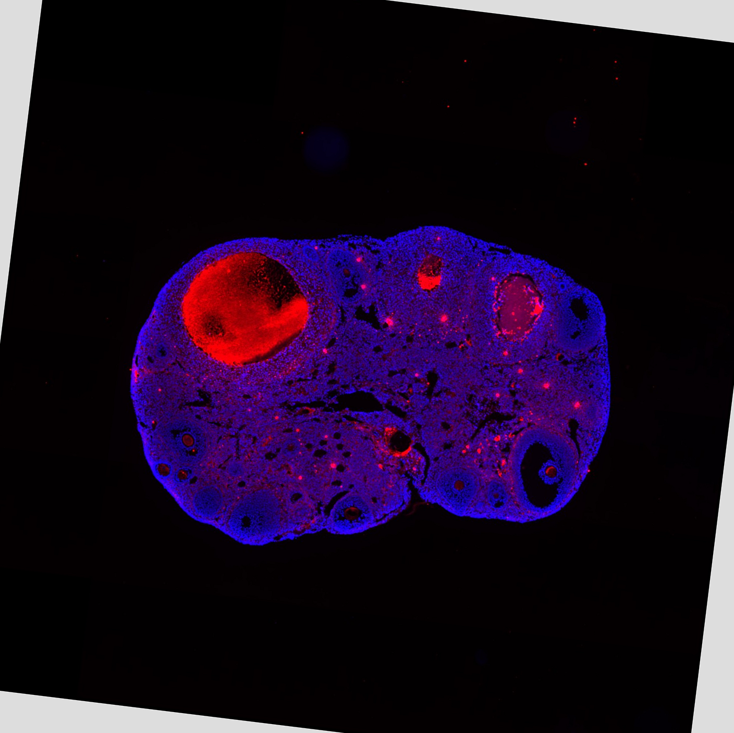

Supplement: Supplementary file 13 — Source data Fig. 6 [file 44321_2025_251_MOESM13_ESM.zip › Figure 6/6F/IL1B DKO LXR.tiff]

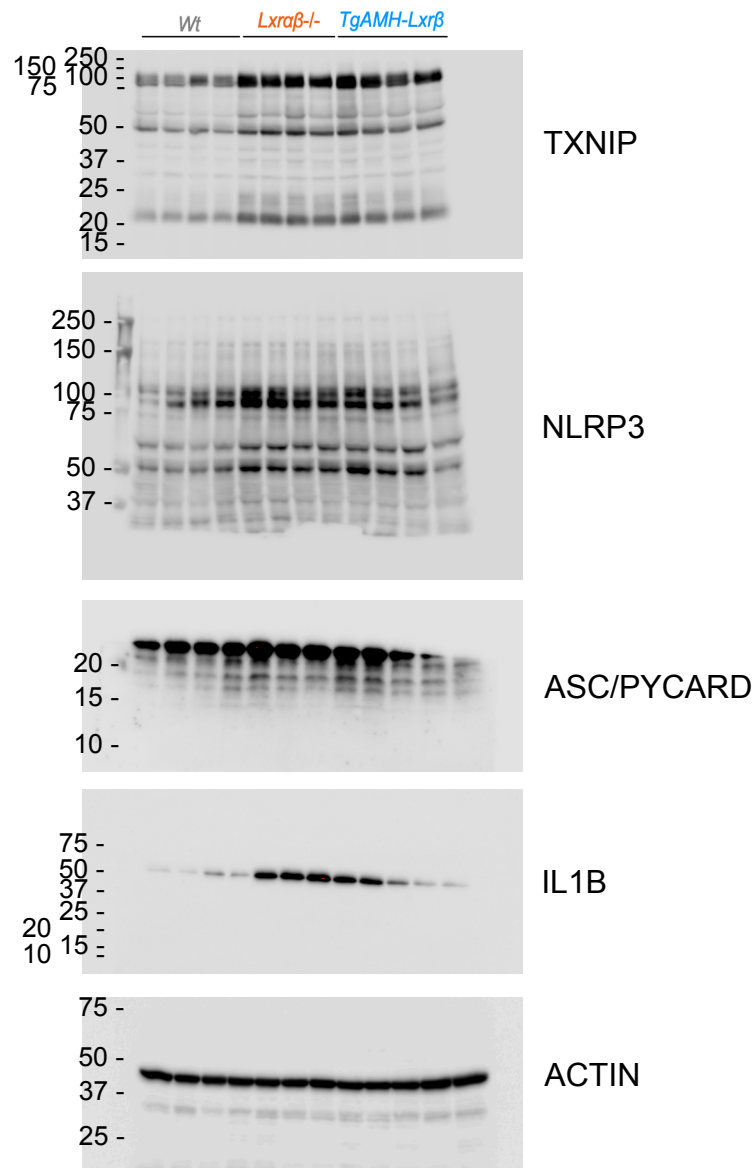

Supplement: Supplementary file 13 — Source data Fig. 6 [file 44321_2025_251_MOESM13_ESM.zip › Figure 6/6C/Western Blots TXNIP NLRP3 ASC-PYCARD IL1B ACTIN.pdf]

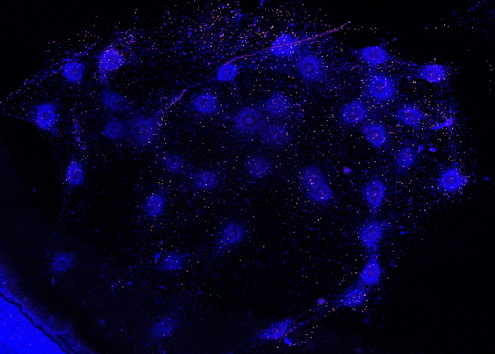

Supplement: Supplementary file 14 — Source data Fig. 7 [file 44321_2025_251_MOESM14_ESM.zip › Figure 7/7H/CD45 COC LXR DKO.tiff]

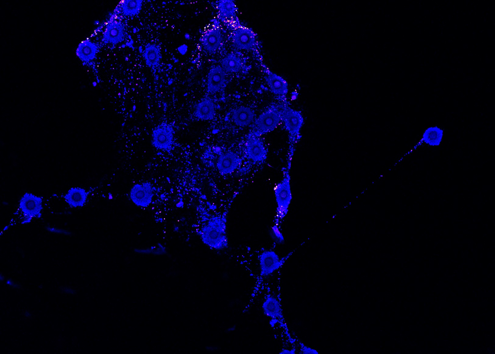

Supplement: Supplementary file 14 — Source data Fig. 7 [file 44321_2025_251_MOESM14_ESM.zip › Figure 7/7H/CD45 COC WT.tiff]

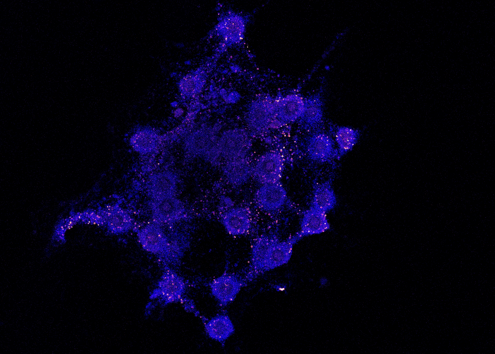

Supplement: Supplementary file 14 — Source data Fig. 7 [file 44321_2025_251_MOESM14_ESM.zip › Figure 7/7H/CD45 COC TG AMH B.tiff]
